# Undergraduate Research Science Capital: Measuring capacity to engage in research

**DOI:** 10.1371/journal.pone.0310053

**Published:** 2024-10-25

**Authors:** Evelyn Abagayle Boyd, Kelly Best Lazar

**Affiliations:** 1 Department of Engineering and Science Education, Clemson University, Clemson, SC, United States of America; 2 Department of Chemistry and Biochemistry, University of Mississippi, University, MS, United States of America; 3 Department of Environmental Engineering and Earth Sciences, Clemson University, Clemson, SC, United States of America; University of Texas Southwestern Medical Center at Dallas, UNITED STATES OF AMERICA

## Abstract

Undergraduate research has been identified as a high-impact educational practice. However, despite the body of evidence on the outcomes of undergraduate research, few studies have focused on the influences students face regarding participation. Developed using Science Capital and Social Cognitive Career Theory, a survey comprised of potential influences to undergraduate science research participation was disseminated to science majors at four R1 institutions in the Southeastern United States. Participation rates across several demographic factors and effect of participation influences were analysed. Results reveal a significantly greater proportion of the Lesbian, Gay, Bisexual, Transgender, Queer, Plus (LGBTQ+) and disability communities indicating participating in research than their peers. Additionally, fourteen participation influences were identified as having a significant difference in their level of influence to the ability to participate in research between researchers and non-researchers. These include professor influence, interest in research, interest in science, coursework in the major, and major all being rated as opportunities with a significant difference of effect between researchers and those who have not yet participated in research. The results of this study will be beneficial for science departments and their respective institutions to improve the equity of access to their undergraduate research experiences.

## Introduction

Undergraduate research experiences (UREs) have a well-documented body of literature supporting their influence on positive student educational outcomes. These experiences have been demonstrated as effective for students in all fields, however they are arguably most prevalent in the sciences [1, 2]. Positive outcomes of UREs for science students specifically include increased student interest in the discipline, increased persistence to graduation with a science degree, clarification of future goals, and increased sense of belonging in the sciences [3–9]. These outcomes have been shown to be amplified for students the longer (or the earlier) in their academic careers that they begin participation in their URE [3, 7, 10]. Despite this information being available to departments, many UREs are not available to students until they are nearing graduation [2]. However, despite the body of literature available supporting the positive outcomes of UREs, few studies have analysed the ways in which students enter UREs. A better understanding of this process will help science departments and their respective institutions improve the equity of access of these highly influential experiences.

### Literature review

#### Types of UREs

There is not a single understanding of what constitutes a URE, however most lead to similar student outcomes. The most common URE in the sciences is apprenticeship-style research in which a student is paired with a research mentor (usually a faculty member or graduate student) and works with them in their research space [2]. These experiences are often at least a semester in length with students participating in research a few hours a week. Another common form of UREs are summer research experiences. Though these are also often apprentice-style in nature, they are usually full time and typically last 8–12 weeks. Course-based undergraduate research experiences, sometimes called CUREs, are another common format for UREs. All CUREs are course-based but not all course-based research experiences are classified as CUREs; for simplicity the CURE acronym will be used to mean all course-based UREs that are incorporated into the curriculum of a class [5]. By nature of being course-based, CUREs are usually one semester, but can sometimes be carried out in multiple semesters as a sequence of courses. Students will always receive course credit for these UREs; however, they are not always clearly labelled as research experiences.

Those three forms of research experiences described above are likely not an exhaustive list of the many forms of research experiences students may encounter but are the three most common in the sciences. Within a specific URE each individual’s experience will likely be different; these individual experiences are further differentiated by institutional differences that impact available opportunities, supports (e.g., major requirements, offices of undergraduate research), and requirements for research participation.

*Outcomes of UREs*. Undergraduate research has been designated a High-Impact Practice by the American Association of Colleges and Universities (AAC&U; [11]). High-Impact Practices (HIPs) are efforts in higher education that have been identified as being especially beneficial for students [1]. With this designation, and calls by professional organizations such as the American Chemical Society (ACS; [12]) and American Physics Society (APS; [13]) to implement UREs into curriculum, many institutions have increased focus on development and assessment UREs. Though this literature review includes many impactful works that contribute to the URE literature, it is not an exhaustive list.

While undergraduate research is beneficial to students in all fields, it is arguably most prevalent in the sciences [1, 14]. A vast body of research has been generated demonstrating the positive outcomes of UREs for science students, including increased student interest in the discipline [8, 10, 15, 16], increased persistence to graduation with a science degree [3–7, 15–17], enhanced career preparation [3, 5, 15, 16, 18–20], clarification of future goals [4, 6, 9, 10, 15, 16, 20–24], improved technical and professional skills [5, 9, 15–17, 22–26], critical thinking gains [3, 15, 25, 27], improved science literacy [3, 5, 15, 25], improved confidence and self-efficacy in science abilities [5, 6, 15, 16, 18, 20–22, 24, 26, 27], and increased sense of belonging in the sciences [6, 7, 15].

These outcomes have been shown to be amplified for students the earlier in their academic careers that they begin participation in their URE, and the longer the duration of their participation in URE(s) [3, 7, 10]. Additionally, benefits are increased for students that are racially/ethnically traditionally underrepresented in STEM [6, 14, 17, 21–23, 25], transfer students [14], and those that have already struggled academically [8]. Despite this information being available to departments, many UREs are not reaching these students who would benefit the most. These students have been found to participate in research experiences at lower rates and for shorter timespans within their college careers [14].

One frequently suggested method of increasing equity of research experiences is to create CUREs [7, 8, 19, 26–28]. Course-based research is a beneficial way to provide research opportunities for a greater number of students than other forms of UREs. Though limited by lab regulations and space, it is possible to fit more students in a CURE lab than can be adequately mentored in an apprenticeship-style research experience. Other suggestions for expanding the availability of UREs include using mentors from industry as opposed to solely university personnel [18] and moving research labs online, which simultaneously increases accessibility and decreases cost [8, 29]. These all help improve the number of students that are able to participate in research but do little to increase student awareness of available opportunities.

#### Opportunities, barriers, and recruitment practices in UREs

Few studies have explored entry into URE participation, and majority of those focused on identifying barriers as a way of improving the process. Haeger et al. [14] included undergraduate students, faculty members, and academic advisors in their URE recruitment study. Many of their identified barriers fall into the categories of institutional barriers (e.g., finding a mentor, fitting it into one’s curriculum), other commitments (e.g., having to use that time for an outside job, familial commitments), and affective concerns (e.g., lack of sense of belonging). Bangera and Brownell [28] described similar barriers but also issues of student awareness regarding URE opportunities, how to pursue them, and the benefits of UREs.

Likewise, Cooper et al. [30] interviewed 85 biology majors and identified ten “rules to research” as suggestions for students to find and secure UREs. Their work especially highlights the hidden curriculum that is often present surrounding research spaces and specifically calls for studies that analyze pathways surrounding UREs with a quantitative approach.

### Theoretical framework

This study lies at the intersection of the individual focus of Science Capital [31] and the institutional focus of Social Cognitive Career Theory (SCCT; [32]). Capital can be generally defined as assets that individuals “carry” with them. If you picture students in a class with backpacks, they may carry backpacks of different styles, brands, or sizes, they may have been purchased or gifted to them from different places or people, and they may be filled with different resources, but they all serve the purpose of helping the student be prepared for class. Likewise, sociological capital are the “things” that we “carry” with us as we are interacting with the world around us. Elements of sociological capital can include, but are not limited to, individual characteristics [e.g., demographic factors], past mentor relationships, and access to resources [33]. Several studies have utilized various conceptualizations of capital to explore UREs [9, 20, 30, 34, 35].

In academic settings, discussions of capital are traditionally primarily applied to arts and humanities settings, Science Capital was developed as a reframing of the sociological capital work of Bourdieu [36] and was designed explicitly to describe how individuals become involved in science experiences and the differing paths they may take to get there. The four major elements of Science Capital were described by Dewitt and colleagues as: *What you know*, *Who you know*, *How you think*, and *What you do* [37]. Combinations of these four elements describe how individuals interact in science contexts.

An important weakness in Bourdieu’s conceptualization of cultural capital is that it carries a deficit approach and views differences in culture as a hierarchical structure. This often leads to assumption that individuals from some cultures “lack” the social and cultural capital required for social mobility. In response to this, Yosso [38] developed the theory of Community Cultural Wealth (CCW). Community Cultural Wealth is related to Bourdieu’s theory and involves all four types of sociological capital (Economic, Symbolic, Cultural, and Social; [36]). However, the focus is on cultural capital with an asset-based mindset. Instead of what do certain cultures lack, the focus is on what each culture teaches the members of their communities, and what other cultures can learn from them. Science Capital was developed after CCW, and Archer et al. [31] described consideration of several reframings of Bourdieu’s capital in its creation to help account for these potential weaknesses.

Social Cognitive Career Theory (SCCT) is frequently used in studies describing the outcomes of undergraduate research participation [21]. Though originally designed to describe career choice, SCCT has been used to understand decision making in a variety of contexts. This theory describes how self-efficacy (an individual’s self-confidence in their ability to accomplish a given task) and outcome expectations (what an individual believes they will come away from a task having accomplished or gained), are influenced by demographic and background factors and play a role in an individual’s career choice [32]. When considering SCCT in undergraduate research settings, it is helpful for understanding how a student’s future goals, such as their career aspirations, may affect their research participation. This leads to the consideration of a fifth area of Science Capital in undergraduate research settings, *How you dream*.

**Fig 1:**
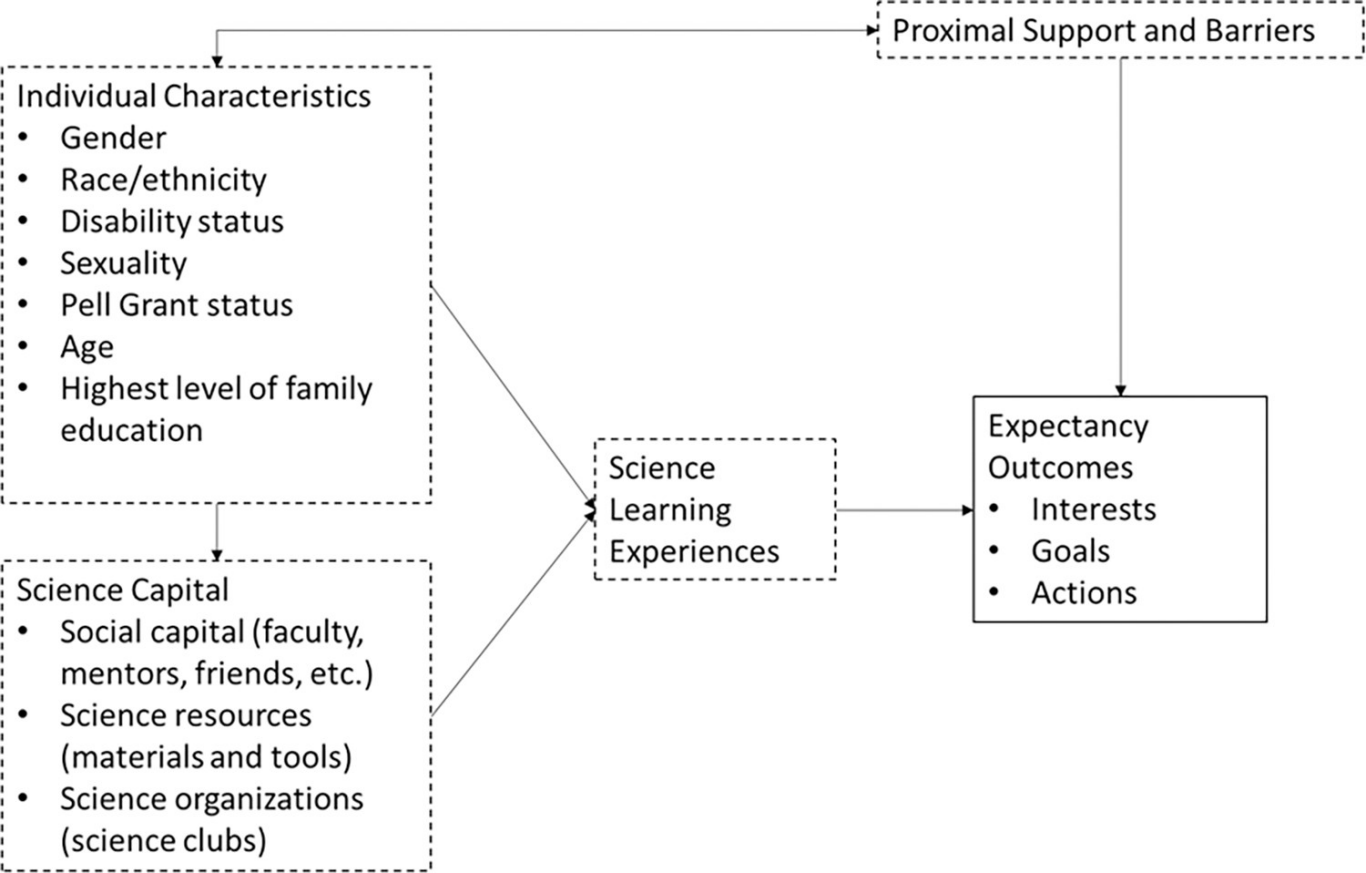
Adaptation of Jones et al model combining science capital and SCCT. Items focused primarily on Science Capital are outlined with dotted lines, items focused primarily on SCCT outlined in solid lines.

Jones et al. [33] developed a model which displays the intersection of Science Capital and SCCT (reproduced in Fig 1). Jones et al. [33] state that their intention of creating their model was to investigate the properties of SCCT constructs that have been shown to contribute to career interests (specifically self-efficacy and expectancy outcomes), as well as components of Science Capital such as prior science experiences, beliefs about the value of science for the future, and family habitus for science (the degree to which the family values science) held by middle school youth when asked about their future career goals. Their model highlights the access that their students have to social capital (e.g., access to mentors and scientists), science resources (e.g., tools and materials), and science-related organizations (e.g., science camp or science museum). They also specifically mention how combining the theories allowed them to consider the support and/or barriers that arise from teachers, family members, and others such as peers and the effect these individuals may have on a student’s decisions. Outcome expectations have been identified as significant components of the academic-career choice process, and are also considered in the creation of this model [32]. The goal of the Jones et al. [33] study was to create an assessment that could measure these constructs for middle school youth and allows researchers to examine how these variables contribute to career aspirations. Additional studies have utilized the Jones et al. [33] model in contexts such as exploring student pathways through technical education majors [39] and comparing the experiences of STEM students in Finland and the United States [40].

Likewise, this study utilized the Jones et al. [33] model as the starting theoretical framework. The model allowed the authors to consider potential influences to participation s and their relationship to UREs. The Jones et al. [33] model combined with factor analysis findings described in our previous work [41] led the authors to consider five areas of Undergraduate Research Science Capital (URSC): *What you know*, what science you know; *Who you know*, science-related Social Capital; *How you think*, which is broadly defined as how you value and understand science; *What you do*, participation in science related activities; and *How you dream*, future science related goals [37, 42].

These five areas of Undergraduate Research Science Capital were used to consider the following research question:

1. What influences are identified by science students as impactful for participation or non-participation in undergraduate research experiences?

## Methodology

### Population

This study is approved exempt from review by Clemson University’s Institutional Review Board (IRB2021-0928). Institutions were selected via a random number generator from a list of public Carnegie Doctoral Universities with Highest Research Activity in the Southeastern United States. One institution is a Hispanic-Serving institution (HSI) while the remaining three are predominately White institutions (PWI). Half of the institutions are land-grant institutions. Electronic surveys were administered at four participating institutions to measure students’ undergraduate research-related science capital. Each participating institution’s IRB approved of participation, and dissemination followed individual institutional guidelines. To reach a broad spectrum of science majors regardless of class standing, and to best control for potential sampling bias, surveys were disseminated via email. Where applicable, department chairs identified potential instructors to assist with survey dissemination. Additionally, science-related clubs and listservs were utilized, and flyers were posted in locations near where science courses meet across campuses (where allowed by institutional guidelines).

Survey responses were collected from April 11, 2022- October 24, 2022. Consent to participate in the study was obtained through a question at the beginning of the survey. Participants were required to indicate they consented to participation to continue the survey. Participant inclusion was determined by those that reported an academic major within the Classification of Instructional Programs (CIP) codes for physical and life sciences [43], a minimum age of eighteen years, and agreeing to the consent documentation presented on the first page of the survey. Upon survey completion, fifty participants were randomly selected to receive a $20 incentive card for their participation; individual instructors were also permitted to offer extra credit for survey completion at their discretion.

An estimated 12,442 students are science majors across the four participating institutions based on publicly available enrollment statistics. One thousand three hundred ninety-five survey responses were obtained resulting in an overall response rate of approximately 11.2%. After completion and inclusion criteria were applied, 833 responses were included in the study resulting in a response rate of approximately 6.7%. Response rates are approximated due to IRB limitations preventing surveys from being distributed to all science majors at all four institutions and exact enrollment statistics are not publicly available. Four hundred fourteen participants (49.7%) completed the survey within their first year of college, 164 (19.7%) in their second year, 124 (14.9%) in their third, 114 (13.7%) in their fourth, and 17 (2.0%) within their fifth year or beyond.

### Population analysis

Due to varying availability of population demographic data for comparison, publicly available data were collected from three different databases [44–46]. As such, it was deemed inappropriate to use one statistical test such as a logistic regression because the population data did not come from one source. Instead, once collected the population proportions were compared to the survey responded proportions using two proportion z-tests to understand the differences between the sample and population proportions and differing groups within the sample. All standard two proportion z-test assumptions were met [47]. Aggregated population demographics across all four institutions are presented in Table 1.

**Table 1:** Representation of different demographic groups within the researcher and non-researcher groups.

Demographic Characteristic	Researcher Proportion (%)	Non-Researcher Proportion (%)	*z* score	*p*-value	Population Proportion (%)	Study Proportion (%)	*z* score	*p*-value
Members of the LGBTQ+^b^ community	22.0	10.3	4.35	< .001^a^	4.9	13.7	-11.66	< .001^a^
Participants with Disability/ies	12.9	6.8	2.86	.004^a^	8.2	8.6	-0.42	.674
Pell Grant recipients	27.4	22.6	1.33	.184	19.4	24.0	3.05	.002^a^
Transfer students	20.3	18.8	-0.34	.728	21.4	19.2	1.53	.276
Students with genders traditionally marginalized in science^c^	73.7	73.6	-0.05	.960	71.4	73.6	-1.39	.226
Students with races/ethnicities traditionally marginalized in science^d^	16.7	20.9	-1.29	.197	21.8	19.7	1.47	.197

Researcher and Non-Researcher proportions are calculated by number of (non-)researchers belonging to category divided by the total number of (non-)researchers and multiplied by 100 to present as a percentage. Percentages are rounded to the tenths decimal place.

^a^ Significant results (p < .05) of the z-test comparing proportions of R and NR groups indicated by an ^a^.

^b^ LGBTQ+ is an abbreviation for Lesbian, Gay, Bisexual, Transgender, Queer plus.

^c^ Students with genders traditionally marginalized in science include female, non-binary, and those who identify with more than one gender.

^d^ Students with races/ethnicities traditionally marginalized in science reported in this study include American Indian or Alaskan Native, Black or African American, Hispanic and/or Latino/a/x, Native Hawaiian or Pacific Islander, and Middle Eastern.

Of the 833 respondents, 240 [28.8%] had participated in research (R) and 593 (71.2%) had not yet participated (NR). Two demographic characteristics exhibited significant differences between the R and NR groups. Researchers had a significantly larger proportion of students (1) identifying as a member of the LGBTQ+ community (22.0% of R, 10.3% of NR; *z* = 4.35, *p* < .001) and (2) self-reporting having a disability (12.9% of R, 6.8% of NR; *z* = 2.86, *p* = .004; Table 1) compared to their peers who had not participated in research. The proportions of the remaining demographic characteristics (first-generation college students, Pell Grant recipients, proportion of transfer students, individuals with traditionally marginalized genders [female, non-binary, and more than one gender selection], and race/ethnicity traditionally underrepresented in science [those reported in this study include American Indian or Alaskan Native, Black or African American, Hispanic and/or Latino/a/x, Native Hawaiian or Pacific Islander, and Middle Eastern] were all statistically similar between the R and NR groups (Table 1).

When asked how many research experiences they had participated in, the majority of the R group had participated in one research experience (54.4%), which was most often in the first year of their college experience (49.1%; Table 2). Approximately 55% of students who participated in research, indicated participation in one or more research experiences outside of their declared major.

**Table 2:** Researcher characteristics (% of researchers).

**Number of research experiences participated in**	
1	54.4
2	28.3
3	10.0
4+	7.4
**Year in college of first research experience**	
1	49.1
2	26.5
3	18.7
4	7.4
5+	0.9
**Type of experiences (select all that apply)**	
Course based	50.0
Lab for credit or pay	43.5
Summer	35.7
Volunteer	17.0

Percentages are rounded to the tenths decimal place.

Student participation changes over time through college. Though half of all research experiences reported by students are course based, lab/research group-based experiences for credit and/or pay become much more common starting in the second year of college (Table 2). Additionally, there were differences in the year in college in which students began participating in research (Table 2); the mean score of several of the participation influences (Interest in Exploring New Ideas, Family Responsibilities, Academic Advisor Influence, Family Influence, Office of Undergraduate Research)was found to be significantly different between first year (beginning of college) and fourth year (nearing end of college) students (S1 Table).

### Description of survey

To measure undergraduate students’ research-related science capital, a survey with both Likert-style and free response questions was administered at all four participating institutions. The survey instrument is available in S1 File. Participants were asked about the number and type of research experiences they had participated in. Additionally, students that identified not having participated in research experiences were asked to identify any reasons for their lack of participation. This item was a multiple-choice, select all that apply question, with the opportunity to fill in free response if the participant desired. Multiple-choice options were identified in the literature and during the survey-vetting process as common barriers to URE participation and included: *“I would prefer to participate in an internship/ Co-op”*, *“I was/ am not aware of research opportunities available to me”*, *“I do not have time in my schedule”*, *“I am not interested in doing research”*, *“I have never considered participating in research”*, *“Research opportunities available to me do not pay well or do not pay at all”*, and/or *“Other*. *Please describe in the textbox*.*”* (S1 File). This item only appeared on the survey for students who identified not having participated in research experiences, so the responses of those who had previously participates in research and left were not captured by this item.

To develop a survey of students’ Science Capital and its influence on URE participation, twenty-five potential influences on participation that were informed by the literature were included on the survey for survey participants to rate on a Likert-type scale from 1 (extremely negative impact)– 7 (extremely positive impact), with a not-applicable option. Not applicable and neutral (4) were two separate response options. Participant instructions for this section of the survey were worded: “*The next questions will help us identify influences that could be considered opportunities or barriers to undergraduate research participation*. *On a scale of (1) Extremely negative impact to (7) Extremely positive impact*, *how much of an impact did the following things have on your ability to participate in undergraduate research*? *Please use NA to indicate any that did not have an effect on you”* (S1 File). Factors that were reported to be a “negative impact” on participation are interpreted to have made research participation more difficult and those reported to be a “positive impact” are interpreted to have made participation easier.

Influences were presented on the survey neutrally so as to not steer the respondents towards opportunity or barrier. Additionally, influences each had a brief description to help clarify meaning and distinguish between items that may seem similar (e.g., “Work-Jobs outside of your research responsibilities”). Skipped/missing influence items were counted as a “NA” response and were not included in analysis for that item. Surveys where the participant skipped a large number of items and did not complete the demographic questions at the end of the survey were counted as incomplete and were completely removed from analysis. The survey was reviewed by educational researchers and undergraduate students prior to dissemination with a focus on pragmatic and communicative validation [48]. In addition to the Likert-style influences, free response questions were included to allow students to include additional influences that may have been missed by the survey and allow for further elaboration. Twenty-three of the twenty-five items were identified from the literature [14, 26]; the influences of COVID-19 and travel to/from research sites were identified as potentially impactful influences that were not previously found in literature and added before survey dissemination. Further details of the survey development phase, including exploratory and confirmatory factor analysis stages, are described in [41].

### Survey analysis

Sample means and standard deviations were calculated from the quantitative survey responses and compared using unpaired *t*-tests to understand the differences between the sample and population proportions and differing groups within the sample. Researchers confirmed that all standard *t*-test assumptions were met before beginning analysis [47]. Free response survey responses (n = 696) were qualitatively coded using magnitude coding by two researchers following protocols described by Saldaña [49]. Magnitude coding is a qualitative analysis technique that allows researchers to consider characteristics such as intensity or frequency while coding the data. It is specifically recommended for instances such as this where qualitative and quantitative data are combined as it assists the researcher in balancing general qualitative guidelines such as “quantity of code does not always equal quality” and frequency of response in large datasets [49]. Intercoder agreement was checked to ensure qualitative coding reliability and found to have a Cohen’s Kappa value of 0.77 [50].

## Results

### Influences to research participation

When asked their primary reason(s) for not participating in research, 68.7% of the NR group reported that they were not aware of available opportunities and 27.1% stated they had never considered research (Table 3). Of all respondents, 68.1% indicated that they hope to participate in research in the future.

**Table 3:** Influences for non-participation in research.

Influences for non-participation in research (Select all that apply; % of non-researchers)	
Not aware of opportunities	68.7
Time	52.9
Prefer to participate in an internship	33.1
Not interested	22.3
Not considered	27.1
Research opportunities do not pay well/at all	7.6

Percentages are rounded to the tenths decimal place.

Mean scores for each influence item on the URSC survey were calculated; these means were used to determine ten opportunities (M≥5.00), nine neutral influences (M = 4.00–4.99), and six influences that typically served as barriers to undergraduate science research experiences (M≤3.90; Table 4).

**Table 4:** Survey responses.

Influence	Sample Mean	Researcher Mean	Non-Researcher Mean	R/NR *p-*value	Cohen’s d
**Opportunities (M≥5.0)**					
Professor Influence	5.11 (1.18)	5.48 (1.14)	4.95 (1.16)	< .001^a^	.46
Interest in Research	5.18 (1.36)	5.56 (1.22)	5.03 (1.39)	< .001^a^	.40
Interest in Science	5.56 (1.16)	5.71 (1.16)	5.49 (1.16)	< .001^a^	.19
Coursework in the Major	5.24 (1.28)	5.44 (1.21)	5.16 (1.29)	.002^a^	.27
Major	5.21 (1.16)	5.37 (1.18)	5.15 (1.15)	.007^a^	.19
Interest in Solving Real World Problems	5.51 (1.13)	5.62 (1.08)	5.47 (1.14)	.113	.24
Interest in Exploring New Ideas	5.46 (1.13)	5.51 (1.19)	5.45 (1.11)	.634	.09
Career Goals	5.59 (1.21)	5.64 (1.18)	5.56 (1.23)	.122	.07
Graduate/Professional School Goals	5.64 (1.22)	5.68 (1.21)	5.62 (1.22)	.440	.09
Interest in Learning New Skills	5.58 (1.13)	5.57 (1.30)	5.58 (1.06)	.710	.01
Interest in Questioning Misconceptions	5.20 (1.23)	5.15 (1.41)	5.22 (1.15)	.518	.06
**Neutral (M = 4.0–4.9)**					
Academic Advisor Influence	4.83 (1.16)	5.06 (1.19)	4.74 (1.13)	< .001^a^	.46
Peer Influence	4.79 (1.15)	5.05 (1.19)	4.68 (1.12)	< .001^a^	.29
Other Mentor Influence	4.72 (1.16)	5.02 (1.13)	4.59 (1.15)	< .001^a^	.65
Awareness of Opportunities	4.19 (1.45)	4.54 (1.46)	4.05 (1.42)	< .001^a^	.34
Family Influence	4.81 (1.22)	5.00 (1.22)	4.73 (1.21)	.001^a^	.22
Finding Opportunities	4.45 (1.37)	4.70 (1.43)	4.35 (1.34)	.004^s^	.26
GPA	4.78 (1.23)	4.95 (1.32)	4.71 (1.18)	.015^s^	.20
Family Responsibilities	4.24 (1.28)	4.38 (1.30)	4.18 (1.27)	.023^s^	.16
Coursework Outside of the Major	4.41 (1.16)	4.56 (1.18)	4.35 (1.15)	.026^s^	.31
Social Responsibilities	4.08 (1.28)	3.98 (1.17)	4.12 (1.32)	.124	.11
K-12 Influence	4.63 (1.27)	4.58 (1.12)	4.65 (1.31)	.479	.06
Office of Undergraduate Research	4.53 (1.15)	4.59 (1.27)	4.50 (1.10)	.312	.10
**Barriers (M≤3.9)**					
Job	3.92 (1.24)	3.87 (1.24)	3.94 (1.24)	.711	.06
Athletics	3.79 (1.20)	3.77 (1.12)	3.80 (1.23)	.727	.03
Religious Responsibilities	3.83 (1.15)	3.94 (1.00)	3.79 (1.20)	.061	.13
COVID-19	3.54 (1.35)	3.54 (1.49)	3.55 (1.29)	.895	.01
Accessibility	3.50 (1.37)	3.61 (1.30)	3.46 (1.39)	.196	.11
Travel	3.90 (1.28)	3.87 (1.23)	3.90 (1.30)	.837	.02

Responses given on a scale of 1 (extremely negative impact)– 7 (extremely positive impact). Influences sorted by Sample Mean response as opportunities (M≥5.0), neutral (M = 4.0–4.99), and barriers (M≤3.9). Standard deviations are shown in parenthesis after each mean.

^a^ Significant results (p < .05) of the t-test comparing R and NR groups indicated by an ^a^.

The three opportunities with the highest mean score were Graduate/Professional School Goals (M = 5.64, SD = 1.22; reported by 59.9% of students), Career Goals (M = 5.59, SD = 1.21; reported by 63.3% of students), and Interest in Learning New Skills (M = 5.58, SD = 1.13; reported by 77.1% of students). The three barriers with the lowest sample means were Accessibility (M = 3.50, SD = 1.37; reported by 18.6% of students), COVID-19 (M = 3.54, SD = 1.35; reported by 32.9% of students), and participation in Athletics (M = 3.79, SD = 1.20; reported by 22.2% of students; Table 4). Students reported on average 12.38 influences as opportunities towards their participation in UREs and 3.01 influences as barriers.

Fourteen influences presented significant differences between the R and NR groups. Of these influences, five were categorized as opportunities (Professor Influence, Interest in Research, Interest in Science, Coursework in the Major, and Major) and nine as neutral influences on average (Academic Advisor Influence, Peer Influence, Other Mentor Influence, awareness of Opportunities, Family Influence, Finding Opportunities, GPA, Family Responsibilities, and Coursework Outside of the Major; Table 4). There was no difference found between mean scores of researchers and non-researchers for influences determined to be barriers to undergraduate research (Table 4).

### Quantitative responses of students with disabilities and LGBTQ+ students

Due to the demonstrated overrepresentation in research participation of students with disabilities and LGBTQ+ students (Table 1), subpopulation analysis was run for each of these student groups. Each survey item factors into one of the five areas of URSC, *What you know*, *Who you know*, *How you think*, *What you do*, and *How you dream* (further description can be found in [41]. The subpopulation analysis was carried out by calculating the average response of the influences in each of the five areas of URSC and comparing the two sub populations to the overall study average using t-tests. Subpopulation results are displayed in Table 5. Lesbian, Gay, Bisexual, Transgender, Queer, plus (LGBTQ+) students indicated influences that fall into the *What you know* form of capital as a significantly greater barrier than their peers. Students with disabilities indicated influences that fall into the *Who you know* form of capital as a significantly greater opportunity than their peers. Finally, both subgroups indicated that influences that fall into *How You Think* as significantly greater opportunities than their peers.

**Table 5:** Subpopulation analysis for LGBTQ+ students and students with disabilities.

	What You Do	Who You Know	What You Dream	How You Think	What You Know
Total Study Average (N = 833)	3.90 (0.75)	4.74 (0.73)	5.22 (1.33)	5.33 (0.98)	4.30 (1.27)
LGBTQ+ Students (N = 142) *p*-value	3.81 (0.73) .185	4.70 (0.67) .542	5.35 (1.54) .294	5.57(0.96) .007^a^	3.97 (1.49) .005^a^
Students with Disabilities (N = 70) *p*-value	3.95 (0.70) .589	4.96 (0.65) < .001^a^	5.79 (1.21) .055	5.70 (1.02) .003^a^	4.38 (1.70) .623

Standard deviations are shown in parenthesis after each mean.

^a^ Significant results (p < .05) of the t-test comparing the subpopulation to the total study average indicated by an ^a.^

### Free response analysis

Replies to four free response questions were coded for additional influences utilizing magnitude coding. Intercoder reliability (K = 0.77) measures were utilized to ensure validity in the coding process. The three most frequently mentioned opportunities leading to research participation are (1) students seeking out research experiences, (2) coursework influences, and (3) professor influences. These influences are in direct opposition to the three most commonly mentioned barriers to research participation: (1) students not knowing how to get involved, (2) the amount of time a research commitment requires, and (3) coursework as a hinderance to participating in research.

Students seeking out research experiences was both the most frequently mentioned opportunity and the most frequently mentioned barrier to research. The majority of the responses that viewed this as an opportunity described how they found research by seeking out experiences on their own, as was the case for this physics major (R), “*I used the [university-wide undergraduate research program] website to learn about research opportunities for students in my major and then I became involved in a group*.*”* On the contrary, the majority of students describing their search for a research experience as a barrier expressed interest in participating but were uncertain how to become involved, as was described by this physics major (NR) “*I would love to participate in research*. *I just don’t know how*. *I haven’t the faintest idea how to begin that process*.”

The effect of coursework (e.g., the time that is required to be dedicated to coursework and opportunities that coursework affords) on students’ participation in UREs was listed among both the most frequent opportunities and barriers. Students, like this geology major (R), described conducting research within courses themselves, “*In Geology*, *we have research classes each semester starting our sophomore year*. *These classes really prepare us to take on our own research*. *We are lucky that the geology program gets us so involved in research so early*.” There were also accounts where courses and curriculum provided a means for students to learn about available opportunities and become involved outside of class.

*“After this one class in which we listened to different people from the department discuss their research, I looked into different people in a research area that interested me. I ended up reaching out to a professor and we wrote a proposal for a project.”-*Physics Major, (R)

However, a biology major (R) described how coursework could be a hinderance to participation, “*My school and work schedules impact my ability to participate in undergraduate research the most*.” Science curricula are often inflexible and outside research experiences may be difficult to schedule while balancing coursework and other outside responsibilities [28].

The third most frequently mentioned opportunity in the free response was professor influence (n = 49). This code consisted of instances where students described being directly invited to participate in research by a professor, or where the student cited a professor as the major influence leading to their research participation. In addition to assisting students, there were accounts of professors inspiring students to want to participate in UREs as was the case for this genetics major (NR), *“My desire to participate in research largely came from the excitement that I saw in my professors in my department*. *I wanted to challenge myself with problem-solving tasks to find solutions to unanswered questions in human medicine*.*”* Despite being a positive influence overall, some respondents (n = 13) shared accounts of a professor creating a negative research environment for them, or their friends dissuading them from participating in a URE. This was the case for a chemistry major (NR) who indicated their largest barrier to participation, *“…not liking how professors treat the undergrads researching with them*, *especially in my major…”*.

### New participation influences

The potential effect of students needing to travel to research sites (M = 3.90, SD = 1.28) and the COVID-19 pandemic (M = 3.54, SD = 1.35; Table 4) were not found as potential influences in the literature but were included in the survey as they were anticipated to have an effect. Travel was supported in the free response by many students mentioning transportation to/from campus or to research sites off campus being a concern. Effects of the COVID-19 pandemic on participation in research was one of the highest barriers to students both by average (M = 3.54, SD = 1.35; Table 4) and prevalence (32.9% of students reporting it as a barrier). This impact was described by many participants, such as this Genetics major:

*“I’ve had some really good experiences with my research advisors and some really negative experiences with some research advisors. I’m not entirely put off by research, but I’ve had two extremes of the spectrum. Also, Covid-19 decimated my opportunities for research in undergrad (fresh-soph years for me) and [I] was completely unable to get anything. This has lead [sic] to a sense of desperation for me to get more experiences before applying to grad school.”*–Genetics Major, (R)

However, several students also described changes in curriculum and research opportunities made due to pandemic response as being an opportunity for them,

*“COVID helped my research opportunities because my ‘big break’ happened after taking a field class that was offered over Spring break in the [Local Research Area] due to travel restrictions. It was here that I met my current research advisor, and he offered me the opportunity to participate in research with him over the summer.”* -Geology Major, (R)

Two other influences that were not anticipated by the research team emerged from the free response section of the survey: mental health considerations (including imposter syndrome) and concerns about citizenship impacting research opportunities. Mental health, appearing as both mental health generally and specific mental health concerns (e.g., imposter syndrome and anxiety), was described as impactful to their non-participation by several students, as exemplified by this Biochemistry major’s reflection.

*“Mental Health as both a[n] outside responsibility to deal with as well as an obstacle for entering research. Having obligations outside of work and school to also take care of mental health in college is time consuming. It’s also an obstacle as my feelings of imposter syndrome and anxiety definitely held me back from participating in research. I often felt unqualified to get involved and the rejection and silence one gets from professors/Pis etc. when first inquiring after research opportunities can be very discouraging especially when dealing with these two issues.”* -Biochemistry Major, (R)

Several other students (n = 15) described mental health concerns as well as imposter syndrome being a barrier to research, both explicitly as with this student and more implicitly as with this NR biology major when asked for the largest barrier to participation, “*I feel I am not competent enough*, *whereas other students are more applicable* [sic] *to doing research*.”

A final new influence was the impact of a student’s citizenship status (indicated by one student as the largest barrier to their participation). Many research opportunities are government funded and require students to be U.S. citizens to participate, particularly if paid. These influences previously missing from the literature provide further insight into the paths students navigate towards their participation in UREs and are beneficial in further developing a scale for URSC.

## Discussion

### Measuring representation within UREs

Significantly larger proportions of research students reported being a member of the LGBTQ+ community (*z* = 4.35, *p* < .001; Table 1) and having a disability (*z* = 2.86, *p* = .004) than their peers who had not previously participated in research. These are encouraging results, perhaps suggesting that members of these two groups are able to overcome persistent messaging about who is (and is not) welcomed into research spaces; additional work is needed to understand the R/NR proportions for these groups [51, 52]. In a national study, Hughes [53] found a disproportionately large number of students that were members of the LGBTQ+ community were also participating in research, however, this engagement did not translate to persistence to a STEM degree. Efforts to support retention of LGBTQ+ students in science majors are often hampered by current data collection and survey methodologies [51, 54]. This is in part because data on these students’ status as members of the LGBTQ+ community is not collected by the majority of nationally representative datasets, and when these data are collected, students may conceal that portion of their identity due to concerns about social acceptance [53, 54]. In response to a 2022 Presidential Executive Order on advancing equality for lesbian, gay, bisexual, transgender, queer, and intersex individuals [55], educational organizations including the American Educational Research Association (AERA) and the American Association for the Advancement of Science (AAAS) have urged the inclusion of sexual orientation and gender identity (SOGI) indicators on surveys conducted by the National Science Foundation (NSF; [56]).

Additionally, the data in this study were self-reported. It is unclear how the self-reporting of these data impacted whether students chose to disclose their disability status on this survey, but it is estimated that as many as two-thirds of students with disabilities do not report them to their universities [57]. This creates a support gap for student accessibility services and under-identification of students reporting a disability in institutional datasets. This also directly affects the construct of “*What you do*” as students are not accessing institutional supports as frequently. Studies on how to support students with identities that may be possible to conceal (e.g., sexuality status, certain disabilities, mental health struggles) have been described by many researchers as a needed avenue of future work [51, 54, 58, 59]. The results of the sub population analysis described by Table 5 reveal potential areas to support LGBTQ+ students and/or students with disabilities entry into UREs. By highlighting the areas these students have identified as particular opportunities and developing ways to assist with lowering the barriers, students may be more likely to be able to participate. Additional discussion of the pathways surrounding UREs described by members of the LGBTQ+ and/or disability communities related to this study can be found in the following works [42, 60].

Citizenship was mentioned by one student in the free response as the largest barrier to their participation. Citizenship status, an often-overlooked equity issue, has been found to significantly affect students’ access to resources and thus their participation in educational opportunities such as UREs [61, 62]. This lack of access to proper resources has major impacts on their *“What you do”* forms of capital. One example of a citizenship barrier is the requirement of several U.S. funding agencies that participants be U.S. citizens to receive their funding. Citizenship status was not explored in the demographic questions, and it is possible that its effect on participants was greater than captured by this analysis.

### Student search for UREs

Students seeking and finding their own research experiences, an influence that draws on students’ “*What you know”* form of URSC, was the most mentioned opportunity in the free response questions for students entering UREs (n = 55). However, it is also the most commonly mentioned barrier in the free response; this is further supported by 68.7% of NR students selecting this as one of the primary reasons they had not yet become involved in research (Table 3). This juxtaposition is exemplified by both influences of finding opportunities (M = 4.45, SD = 1.37) and awareness of opportunities (M = 4.19, SD = 1.45; Table 4) presenting a neutral sample mean, indicating that it was an opportunity for some students, but a barrier for others. Science instructors and their departments could help mitigate this barrier by communicating with their students what opportunities are available, and how to access them, early and often. While this work did not seek to determine the primary method of communication from departments to students about research opportunities, we suggest that this communication be multi-modal (i.e., not just through email) and scaffolded within courses, with explicit directions on how to find more information. The approach will likely decrease the barriers between students and research experiences and help all students find their place in research spaces but will especially benefit those students with lower amounts of URSC.

In this study, respondents indicated that CUREs were the most common research experience overall, especially for first year students (Table 2). However, other forms of research experiences become more common as students advanced through their collegiate careers, indicating that as students’ progress through college and gain more science capital, the forms of UREs they participate in also shift (Table 2). Studies have suggested increasing the incorporation of research experiences into courses to increase availability of opportunities and participation, and has been found to be a beneficial way to provide research opportunities for a greater number of students than other forms of UREs [7, 8, 19, 26–28]. Though limited by lab regulations and space, it is possible to fit more students in a CURE lab than can be adequately mentored in a traditional apprenticeship-style research experience. Additionally, by providing opportunities for research and coursework simultaneously, CUREs can assist with many of the commonly mentioned barriers to URE participation, including students searching for opportunities, the amount of time research takes, and coursework preventing students from participating in research (Table 4). Bingham [58] found that significantly fewer students viewed logistics (including travel, a newly added barrier in our study) as a barrier to URE participation when the research was course-based. Students interact with course material daily and it is a convenient means of sharing information about opportunities such as UREs. However, when implementing CUREs it is important to ensure adequate support for the faculty members developing the CURE as the development process is often different and requires more time to prepare than traditional courses [29].

### Professor influence

Instructors often serve as mentors to their students both inside and outside of their courses and contribute to their “*Who you know*” capital. The third most frequently mentioned opportunity in the free response was professor influence (n = 49). Additionally, 65.6% of students quantitatively responded with this as an opportunity (M = 5.11, SD = 1.18; Table 4). The term “professor” was utilized in the survey as it was identified as the clearest description of a instructor by the undergraduates that participated in the survey vetting process, it is not intended to exclude instructors of other titles or ranks within higher education. Haegar et al. [14] found that professor influence was the most common opportunity for finding research experiences and reported no negative professor interactions influencing research participation at the midsized institution of study. The differences could be attributed to considering different influences into research and/or the difference in size between the institutions leading to less opportunity for individualized professor interactions.

Research experiences have the potential to be unhappy and unsafe environments for any student, yet those who are members of communities underrepresented in science, commonly based on gender, race/ethnicity, sexuality, and those with disabilities, are particularly vulnerable [63–65]. Students’ relationship with professors as their research advisors greatly impacts the student experience and research group environment [59, 66]. It is important that institutions acknowledge the potential for these situations to become negative and proactively implement guidelines to ensure that UREs are a safe and positive environment for students [67, 68].

### Accessibility & student health

How students access and interact with their UREs are described by their *“What you do*” capital. Accessibility was found to have the lowest sample mean, indicating that students viewed it as the greatest barrier to their participation in UREs (M = 3.50, SD = 1.37; Table 4). A science curriculum often requires long labs and field environments that can be difficult to navigate for any student, but can be especially challenging for those students with accessibility needs [69, 70]. Similarly, the impact of student mental health on participation in research, a newly identified influence in this study, is a related understudied area. Cooper et al. [59] have explored student depression and its effect on persistence in UREs as well as a student’s relationship with their research advisor. They found that students’ depression negatively affected their motivation to participate in UREs and their engagement and productivity while participating [59]. Though coded separately from mental health, students also described imposter syndrome as a barrier to participation. In science fields, imposter syndrome has been found to be more prevalent in highly achieving students, women, and members of traditionally marginalized racial, ethnic, and religious groups [71].

Lastly, the COVID-19 pandemic has left a lasting impact on science and higher education as a whole [72, 73]. Engaged learning opportunities such as UREs are not exempt from these effects [74, 75]. Students reported that complications of the COVID-19 pandemic resulted in one of the highest barriers to student participation in research both by mean score (M = 3.54, SD = 1.35; Table 4) and prevalence (32.9% of students reporting it as a barrier). However, some students did describe how curricular changes made due to COVID were an opportunity for them to engage in research (see *Free Response Analysis* in Results section above). As institutions continue to adapt, there are lessons that can be learned from the pandemic response that can improve the accessibility of these experiences for students in the future, including the possibilities of online UREs [76] and considering field research sites that are closer to campus to limit travel needs. Ensuring the accessibility of UREs is an important consideration for departments in an effort to make science available to all [58, 77, 78].

### URE impact on science recruitment and retention

It is also of note that a majority of students who participated in research (~55%) indicated participation in one or more research experiences outside of their declared major. Undergraduate research experiences have been closely linked with students’ major choice and persistence to graduation as well as their likelihood of entering the STEM workforce [6, 16]. Additionally, the benefits of UREs have been found to cross research experience types and disciplines [2]. This could mean that for students in disciplines with large enrollments (that struggle finding space and resources to meet the needs of all students) can seek out opportunities in other disciplines that have the potential to transfer research and critical thinking skills. Encouraging students to pursue UREs outside of their immediate discipline could expose them to other ideas within science which could lead to further solidification of major choice and career goals.

## Limitations

Study recruitment varied slightly between institutions to comply with each institution’s IRB requirements. As such, the extent of sampling bias at some of the institutions may vary. The study sample has an over-representation of life science students compared to the national population, additionally, there was an over-representation of Pell-Grant recipients, and students with disabilities. These demographic factors could have influenced the overall results and findings.

Additionally, this study data is not generalizable beyond Public R1 institutions in the Southeastern United States. The study was limited to one geographic region to improve the generalizability in that region, however the experiences of students at other institution types such as Historically Black Colleges and Universities (HBCUs), smaller institutions, or institutions in different geographic regions may not be well represented by these findings.

## Conclusion

**Fig 2:**
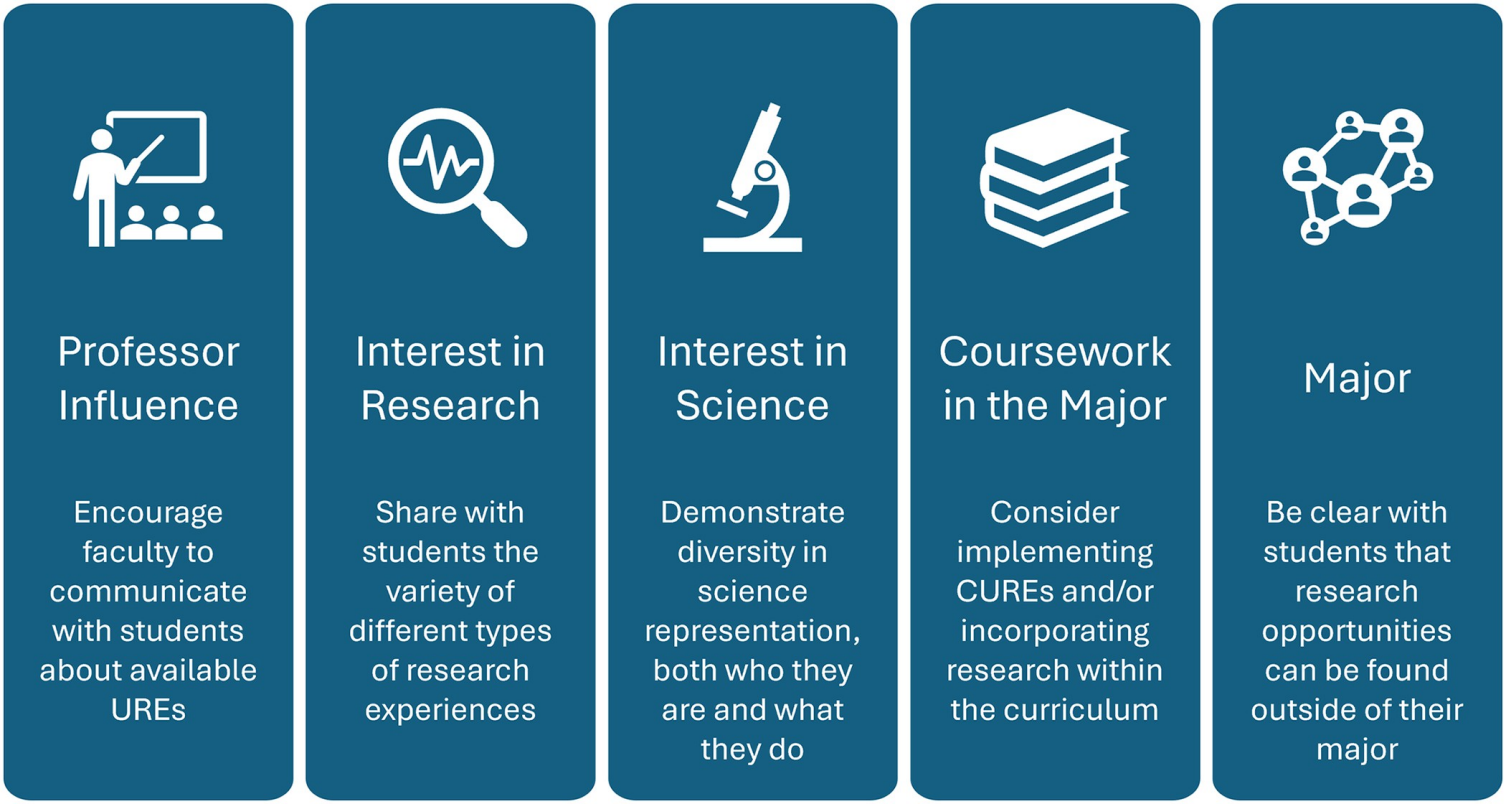
Considerations for promoting opportunities to UREs.

Previous studies have identified four main areas of Science Capital: *What you know*, *Who you know*, *How you think*, and *What you do* [37]. When considering educational practices such as UREs, the effect of students’ goals on their participation is an additional important area of consideration. This led to the inclusion of the SCCT-influenced construct, *What you dream*, to the conception of URSC. The combination of these five factors captures many of the influences that contribute to student participation (or non-participation) in UREs. The majority of the most common and strongly identified opportunities and barriers into research experiences are related to science capital, including finding research experiences, time and the influence of coursework on research participation. This holds true for the influences that are significantly different between the R and NR groups and provides further evidence towards the conceptualization of URSC. Fig 2 displays the five opportunities identified by this study to have a significant difference between the R and NR groups along with suggestions of ways to promote these opportunities so that UREs may be more accessible for all students. There are many ways to participate in research experiences, but by increasing students’ capital and raising awareness of research opportunities and the potential benefits of participating in them, students can make informed decisions about their future research participation.

## Supporting information

S1 File Undergraduate Research Science Capital survey. (DOCX)

S1 Table Comparison of 1^st^ year and 4^th+^ year student responses to Undergraduate Research Science Capital scale items. Asterisks indicate statistical significance.
(DOCX)
