# Supplementary material for: Undergraduate Research Science Capital: Measuring capacity to engage in research
Source: PLoS One. 2024 Oct 25;19(10):e0310053. doi: 10.1371/journal.pone.0310053 (PMC11508476; doi:10.1371/journal.pone.0310053)
Supplement: S1 File — (DOCX) [file pone.0310053.s001.docx]

Supporting Information 1: Undergraduate Research Science Capital Survey

 The Council on Undergraduate Research (CUR) defines undergraduate research as:

"A mentored investigation or creative inquiry conducted by undergraduates that seeks to make a scholarly or artistic contribution to knowledge."

Undergraduate research experiences can be any research activities you have participated in your time in college. These are often either in classes (sometimes called CURES); for course credit and/or pay with a professor, graduate student, or other mentor; or summer experiences (sometimes called REUs). The next few questions will ask about your undergraduate research experiences. If you have not had any undergraduate research experiences, please respond as such.

What year in college were you when you started participating in research for the first time? (If you started in the summer, then please select the next college year)

o 1st

o 2nd

o 3rd

o 4th

o 5th+

o I have not participated in research yet, but I plan to in the future.

o I have not participated in research yet and I do not plan to.

Including any you are participating in currently, how many research experiences have you had?

o 0

o 1

o 2

o 3

o 4+

What kind of experiences were they (Select all that apply)? *(Only displayed for students who indicated previous research participation)*

▢ Course-based (research in a class)

▢ Summer

▢ In a lab for credit or pay (not during the summer)

▢ Volunteer (no course credit or pay)

▢ Other. Please describe in textbox

On a scale of 1 (Not at all interested) - 5 (Extremely interested), how interested are you in participating in undergraduate research? .*(Only displayed for students who HAD NOT indicated previous research participation)*

o 5 - Extremely interested

o 4 - Interested

o 3 - Neutral

o 2 - Not interested but might change mind

o 1 - Not at all interested

Which of the following has influenced your lack of participation in undergraduate research? Please select all that apply.*(Only displayed for students who HAD NOT indicated previous research participation)*

▢ I would prefer to participate in an internship/ Co-op.

▢ I was/ am not aware of research opportunities available to me.

▢ I do not have time in my schedule.

▢ I am not interested in doing research.

▢ I have never considered participating in research.

▢ Research opportunities available to me do not pay well or do not pay at all.

Other. Please describe in the textbox

Do you hope to/ have plans to participate in any research experiences in the future?

o Yes

o No

o Unsure/ prefer not to answer

The next questions will help us identify influences that could be considered opportunities or barriers to undergraduate research participation. On a scale of (1) Extremely negative impact to (7) Extremely positive impact, how much of an impact did the following things have on your ability to participate in undergraduate research? Please use NA to indicate any that did not have an effect on you.

**Outside responsibilities – Responsibilities that may influence your ability to participate in undergraduate research.**

NA 1 Extremely Negative 2 Very Negative 3 Negative 4 Neutral 5 Positive 6 Very Positive 7 Extremely Positive

- Family obligations - Family can be biological or chosen. (e.g., care responsibilities, driving family members places, etc.)
- Work-Jobs outside of your research responsibilities
- Athletics – School sponsored athletic obligations (NCAA, intramural, club, etc.)
- Religious obligations
- Social obligations – Activities outside of those already mentioned that may influence your ability to participate in undergraduate research (e.g., Greek life, clubs, friends)
- Other (Please describe)

**Influential people - Interactions with others that may influence your participation in undergraduate research.**

NA 1 Extremely Negative 2 Very Negative 3 Negative 4 Neutral 5 Positive 6 Very Positive 7 Extremely Positive

- Professors – Interactions in or outside of class
- Teachers (from K-12)
- Academic Advisors - Interactions in or outside of official advising time
- Other Students
- Office of Undergraduate Research - If your school has one, if not, or you don’t know, mark NA
- Family Members – Family can be biological or chosen
- Other Mentors- Anyone you consider a mentor that has not been previously listed
- Other (Please describe)

**Courses – Classes that may influence your participation in undergraduate research (for example, requirements to graduate or courses whose content influenced your abilities to participate.)**

NA 1 Extremely Negative 2 Very Negative 3 Negative 4 Neutral 5 Positive 6 Very Positive 7 Extremely Positive

- Major Courses – classes within your major(s)
- Other courses outside of major(s)

**Future goals - Goals that may influence your decision to participate in undergraduate research.**

- NA 1 Extremely Negative 2 Very Negative 3 Negative 4 Neutral 5 Positive 6 Very Positive 7 Extremely Positive
- Career Goals
- Grad/ Professional School Goals
- Other (Please describe)

**Interest - Your interest in participating in undergraduate research**.

 NA 1 Extremely Negative 2 Very Negative 3 Negative 4 Neutral 5 Positive 6 Very Positive 7 Extremely Positive

Interest in research

- Interest in science generally
- Interest in solving real world problems
- Interest in exploring new ideas
- Interest in learning new skills
- Interest in questioning misconceptions

**Opportunity - Impacts on your ability to participate in research experiences.**

NA 1 Extremely Negative 2 Very Negative 3 Negative 4 Neutral 5 Positive 6 Very Positive 7 Extremely Positive

- Finding a research opportunity
- Awareness of research opportunities
- Your GPA
- Your Major
- COVID-19 – Anything COVID related in the past or current (e.g., research being online, restrictions in place because of COVID)
- Disability Limitations – Any disability you identify with
- Travel - Transportation to and from research locations

Free Response Questions:

- Would you like to share more details about any of your above responses?
- Are there any other influences that impacted your interest and/ or ability to participate in undergraduate research? If so please describe them and the extent to which they had an impact. This is to capture any influences that may have been previously missed in the survey.
- How did you become involved in undergraduate research? Please describe*. (Only displayed for students who indicated previous research participation)*
- What are the major reasons that contributed to you not participating in undergraduate research? Please describe. *(Only displayed for students who HAD NOT indicated previous research participation)*
