# Supplementary material for: Undergraduate Research Science Capital: Measuring capacity to engage in research
Source: PLoS One. 2024 Oct 25;19(10):e0310053. doi: 10.1371/journal.pone.0310053 (PMC11508476; doi:10.1371/journal.pone.0310053)
Supplement: S1 Table — Asterisks indicate statistical significance. (DOCX) [file pone.0310053.s002.docx]

| **Influences** | **Researchers** | | | | | | **Non-Researchers** | | | | | |
| --- | --- | --- | --- | --- | --- | --- | --- | --- | --- | --- | --- | --- |
|  | ***p*-value** | **Cohen’s d** | **1^st^ Year Mean** | **SD** | **4^tth+^ Year Mean** | **SD** | ***p*-value** | **Cohen’s d** | **1^st^ Year Mean** | **SD** | **4^tth+^ Year Mean** | **SD** |
| Professor Influence | .062 | -.282 | 5.13 | 1.22 | 5.53 | 1.09 | .272 | -.098 | 4.95 | 1.17 | 5.07 | 1.18 |
| Major | .110 | -.221 | 5.15 | 1.21 | 5.40 | 1.09 | .502 | -.406 | 5.11 | 1.16 | 5.23 | 1.26 |
| Interest in Research | .526 | .239 | 5.36 | 1.03 | 5.50 | 1.31 | .072 | .037 | 5.06 | 1.34 | 4.59 | 1.72 |
| Interest in Science | .115 |  | 5.44 | 1.15 | 5.77 | 1.23 | .886 | -.321 | 5.46 | 1.81 | 5.49 | 1.26 |
| Interest in Solving Real World Problems | .083 | .041 | 5.37 | 1.15 | 5.71 | 1.02 | .149 | -.520 | 5.38 | 1.15 | 5.63 | 1.19 |
| Interest in Exploring New Ideas | .002* | -.198 | 4.98 | 1.28 | 5.63 | 1.06 | .329 | -.448 | 5.41 | 1.10 | 5.57 | 1.21 |
| Career Goals | .344 | .514 | 5.68 | 1.05 | 5.48 | 1.28 | .540 | -.217 | 5.60 | 1.20 | 5.48 | 1.36 |
| Graduate/Professional School Goals | .417 | .206 | 5.49 | 1.25 | 5.67 | 1.18 | .041* | .339 | 5.27 | 1.56 | 5.06 | 1.34 |
| Interest in Learning New Skills | .017 | -.082 | 5.06 | 1.47 | 5.66 | 1.22 | .385 | -.436 | 5.52 | 1.05 | 5.67 | 1.19 |
| Interest in Questioning Misconceptions | .182 | -.165 | 4.92 | 1.41 | 5.16 | 1.47 | .818 | -.036 | 5.21 | 1.13 | 5.25 | 1.25 |
| Family Responsibilities | .007* | .090 | 4.33 | 1.44 | 4.21 | 1.28 | .313 | -.160 | 4.19 | 1.40 | 4.41 | 1.45 |
| Academic Advisor Influence | .005* | .160 | 5.35 | 1.29 | 4.74 | 1.05 | .366 | .099 | 4.75 | 1.11 | 4.59 | 1.14 |
| Peer Influence | .362 | -.192 | 5.13 | 1.43 | 5.93 | 1.06 | .752 | -.369 | 4.59 | 1.10 | 4.65 | 1.17 |
| Family Influence | .007* | .137 | 5.27 | 1.06 | 4.68 | 1.27 | .055 | -.007 | 4.76 | 1.23 | 4.37 | 1.36 |
| Other Mentors | .095 | .704 | 5.23 | 1.24 | 4.85 | 1.09 | .313 | -.373 | 4.59 | 1.12 | 4.63 | 1.05 |
| GPA | .494 | -.123 | 4.96 | 1.37 | 5.13 | 1.27 | .841 | .031 | 4.21 | 1.39 | 3.65 | 1.59 |
| Social Responsibilities | .220 | .334 | 4.27 | 1.16 | 3.87 | 1.22 | .010* | .399 | 4.72 | 1.42 | 5.11 | 1.16 |
| K-12 Influence | .210 | .166 | 4.63 | 1.39 | 4.42 | 1.16 | .011* | .429 | 4.70 | 1.38 | 4.13 | 1.11 |
| Office of Undergraduate Research | .010* | .123 | 4.88 | 1.18 | 4.24 | 1.27 | .533 | -.262 | 4.55 | 1.09 | 4.41 | 1.45 |
| Job | .224 | .230 | 3.98 | 1.07 | 3.69 | 1.41 | .220 | -.197 | 3.86 | 1.33 | 4.13 | 1.75 |
| Athletics | .082 | .387 | 3.75 | 1.01 | 3.28 | 1.39 | .786 | =.048 | 3.73 | 1.72 | 3.81 | 1.41 |
| Religious Responsibilities | .087 | .385 | 4.03 | 1.42 | 3.54 | 1.10 | .702 | -.068 | 3.62 | 1.41 | 3.72 | 1.52 |
| COVID-19 | .029 | .026 | 3.87 | 1.29 | 3.27 | 1.59 | .098 | .320 | 3.66 | 1.24 | 3.25 | 1.54 |
| Accessibility | 1.00 | -.483 | 3.52 | 1.28 | 3.52 | 1.37 | .963 | -.010 | 3.42 | 1.43 | 3.43 | 1.20 |
| Travel | .367 | -.207 | 3.87 | 1.36 | 3.64 | 1.21 | .537 | .103 | 3.94 | 1.27 | 3.80 | 1.55 |

Supporting Information Table 1: Comparison of 1^st^ year and 4^th+^ year student responses to Undergraduate Research Science Capital Scale items. Asterisks indicate statistical significance.
